# Iron Deficiency in Heart Failure: A Practical Guide

**DOI:** 10.3390/nu5093730

**Published:** 2013-09-23

**Authors:** Nicole Ebner, Stephan von Haehling

**Affiliations:** 1 Applied Cachexia Research, Department of Cardiology, Charité Medical School, Campus Virchow-Klinikum, Berlin 13353, Germany; E-Mail: nicole.ebner@charite.de; 2 Center for Cardiovascular Research (CCR), Charité Medical School, Campus Mitte, Berlin 10117, Germany

**Keywords:** heart failure, iron deficiency, anaemia, metabolism

## Abstract

Iron is an element necessary for cells due to its capacity of transporting oxygen and electrons. One of the important co-morbidities in heart failure is iron deficiency. Iron has relevant biological functions, for example, the formation of haemoglobin, myoglobin and numerous enzymatic groups. The prevalence of iron deficiency increases with the severity of heart failure. For a long time, the influence of iron deficiency was underestimated especially in terms of worsening of cardiovascular diseases and of developing anaemia. In recent years, studies with intravenous iron agents in patients with iron deficiency and cardiovascular diseases indicated new insights in the improvement of therapy. Experimental studies support the understanding of iron metabolism. Many physicians remain doubtful of the use of intravenous iron due to reports of side effects. The aim of this review is to describe iron metabolism in humans, to highlight the influence of iron deficiency on the course and symptoms of heart failure, discuss diagnostic tools of iron deficiency and provide guidance on the use of intravenous iron.

## 1. Introduction

Iron is an essential trace element present in a number of molecular systems, and it is increasingly recognized as an important co-factor for a variety of cell systems [1]. It has been acknowledged that iron plays an important role in oxygen transport as well as in cell growth and proliferation, mostly because it is unique in its ability to be present in either a reduced or an oxidized state. In recent years, more insight has been gained into iron physiology and the regulation of cellular iron homeostasis. Iron deficiency occurs, for example, when the dietary intake is inadequate, during times of digestive blood loss or menstrual periods, or during states that excessively increased iron requirements, particularly during childhood growth or pregnancy [2]. In patients with chronic illnesses, on the other hand, iron may become immobilizable as a consequence of chronic inflammation thus leading to a state termed functional iron deficiency. Recent studies have shown that iron deficiency is very common in patients with heart failure (HF) [3,4,5], and its prevalence increases with increasing New York Heart Association (NYHA) class. Even though a large meta-analysis of major HF trials has shown that the prevalence of anaemia is around 37% in all patients with HF [6], iron deficiency has important prognostic [7] and quality-of-life implications [8] irrespective of the presence of anaemia. Iron deficiency, whether absolute or functional, is a frequent finding in HF patients presenting with anaemia as well, affecting up to 80% of these individuals [9].

In humans, intracellular iron is stored as ferritin and, in times of iron overload, as haemosiderin whose excessive presence in macrophages is responsible for the color of haematoma. Values of serum ferritin are a clinically meaningful proxy to reflect body iron stores as this protein spills over from intracellular stores into the bloodstream. Ferritin, however, is also an acute phase reactant whose levels may increase during inflammatory processes. Transferrin saturation reflects the relative amount of transferrin that is loaded with iron. In contrast to ferritin, transferrin is a negative acute phase reactant, *i.e.*, its levels decrease during times of inflammation. Importantly, neither serum iron nor serum transferrin alone should be used as indicators of iron status [10]. In addition, it is important to understand that different cut-off values indicate iron deficiency in healthy subjects and in patients with chronic illness. Serum ferritin values normally given in laboratory print-outs reflect the cut-off values in healthy humans, and the laboratory is usually unaware of the presence of a chronic illness, particularly of chronic HF, chronic kidney disease, or cancer. The purpose of this review article is to give an overview of iron metabolism. Additionally this review provide insight into clinical effects of iron deficiency in patients with chronic HF, to discuss diagnostic tools of iron deficiency and to provide guidance on the use of intravenous (IV) iron preparations.

## 2. Iron Metabolism

**Figure 1:**
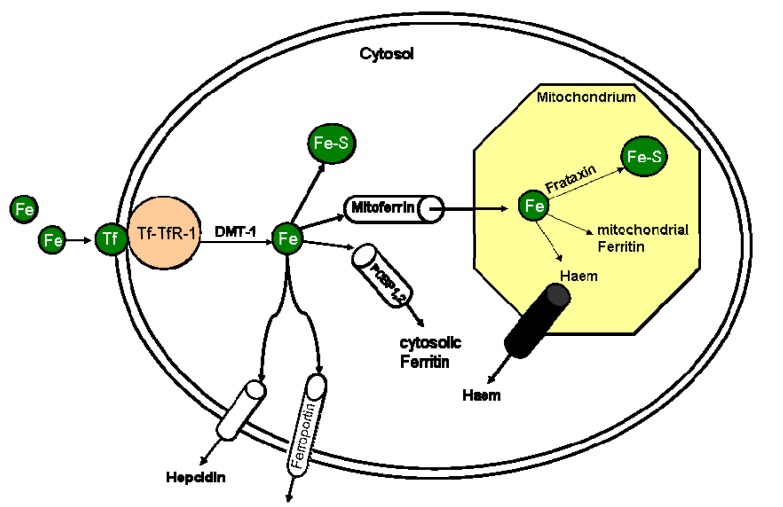
Overview of the cellular iron metabolism.

Iron deficiency is a major reason for the development of anaemia [9]. Patients with chronic HF develop an iron deficit by gradual dismantling of their iron stores, by faulty iron absorption, or by the reduced ability to mobilize iron “trapped” inside the reticuloendothelial system [11]. To develop an understanding of the role of iron in HF, it is important to understand the mechanisms of iron absorption and distribution; in the strictest sense of the word, iron is not metabolized but rather its absorption and distribution are tightly regulated. However, there are two different pathways of iron absorption, one for haem-iron across a haem transporter and another for iron in its ferrous form, known as Fe(II). The latter transport mechanism is called divalent metal transporter (DMT) [2]. Iron absorption across the gut wall is only possible in the ferrous form, *i.e.*, as Fe(II), and ferric iron or Fe(III) that is found in vegetables needs to be reduced to Fe(II) before absorption. Many nutritional factors influence iron absorption. Phytates, for example, contained in a great number of grains and fibers form poorly soluble complexes with iron, thereby reducing its absorption [2]. Polyphenols present in tea, coffee, wine and legumes also interfere with iron uptake. Phosphates and phosphoproteins contained in egg yolk and milk also reduce its absorption [2]. The cytosolic protein that accumulates iron is ferritin. Ferritin protects cells from iron toxicity and prevents iron from reacting with other cellular constituents; in addition, it allows for its controlled release according to cell requirements [2]. Under normal conditions, nearly the total amount of circulating iron is transported by transferrin [2]. The amount of iron transported in serum ferritin is low compared with transferrin-iron. Cohen *et al.* suggested therefore that serum ferritin is not a major pathway of systemic iron transport, but locally secreted ferritin may play such a role in selected tissues [12]. Figure 1 gives an overview of cellular iron uptake and distribution. When iron enters the bloodstream, ferroxidases oxidize Fe(II) to Fe(III) and facilitate its binding by transferrin. 

Iron binds to transferrin (Tf) and interacts with transferrin receptor 1 (TfR-1). This Tf-TFR-1 complex is internalized by the cell, constituting an endosome. Endosomes are acidified by proton uptake, this allowing the release of iron from the endosome into the cytosol by divalent metal transporter (DMT-1). Cytoplasm iron can be used for iron-sulfor-cluster (Fe-S) synthesis or stored within cytosolic ferritin facilitated by Poly-(C) Binding Protein 1 and 2 (PCBP-1,2) chaperone or can be transported into mitochondrium via mitoferrin (Mfrn). Mitochondrial iron can be used for haem synthesis, stored within mitochondrial ferritin or for iron-sulfor-cluster (Fe-S) synthesis.

Besides acting as an iron shuttle in the bloodstream, transferrin is also the major component of iron transport from the bloodstream into all body cells and tissues. Each transferrin molecule is able to bind two atoms of Fe(III) [13]. Transferrin molecules which transport one or two iron atoms bind to specific transferrin receptors (TfRs) on the cell surface. Two different forms of transferrin receptors (TfR-1 and TfR-2) are known, which are differently regulated and expressed [11]. TfR-1 is expressed on all cell types and TfR-2 is mainly expressed in liver and erythroblasts [11]. Serum iron binds to transferrin and, upon reaching its target cell, interacts with TfR-1 [14]. The transferrin/TfR1-complex is concentrated in clathrin-coated pits on the cell surface and undergoes endocytosis [15]. A proton-pumping adenosine triphosphate (ATP)-ase lowers the pH of the endosome eventually yielding iron release [13]. Released iron is exported into the cytosol by the divalent metal transporter. The reduction of Fe(III) to Fe(II) is followed by the transport of iron from the endosome inside the cytosol. Cytoplasmic iron can then be used to assemble iron-sulphur-clusters (Fe-S), or it can be transported via mitoferrin into mitochondria where iron is an essential element in the enzymes of the respiratory chain [16]. Cytosolic iron can also be stored inside of ferritin.

Even though some insight has been gained in recent years, pathways that enable iron trafficking from the endosome to the mitochondrium and to other cellular sites are not well understood [13]. Generally, a large number of proteins need iron as a co-factor, and the two major elements that require iron are haem and Fe-S clusters [13]. Under iron replete conditions, iron regulatory protein-1 (IRP1) binds an Fe-S cluster and acts as an cytosolic aconitase when Fe-S cluster synthesis is normal. The presence of Fe-S-cluster determine the function of the aconitase. Aconitase is an essential enzyme in the citrate cycle that catalyzes the reaction from citrate into aconitate and needs Fe-S cluster as cofactor. When cellular iron levels are low, IRP loses aconitase activity due to reduction in Fe-S cluster synthesis [15]. Iron deficiency triggered reduction in Fe-S cluster results in a reduced production of ATP during citrate cycle and therefore results in reduced exercise capacity and fatigue. Defective Fe-S cluster synthesis causing both mitochondrial iron overload and cytosolic iron deficiency [14]. Under physiological conditions, iron transport is highly conserved and is controlled via negative feedback regulatory mechanisms involving transferrin and its receptors as well as other iron transporters. In several clinical conditions, including primary haemochromatosis and secondary iron-overload, iron metabolism is disturbed, which, in combination with modifying environmental factors, leads to chronic iron overload [17]. In iron-overload conditions, iron in the circulation typically exceeds the serum transferrin iron-binding capacity, leading to the appearance of nontransferrin-bound iron, which is highly reactive [18].

## 3. Iron Deficiency in Health and Disease

From a practical viewpoint it is important to understand that iron deficiency can be classified as absolute or functional. Absolute iron deficiency implies depleted iron stores, functional iron deficiency means that iron is present in the human body, but it cannot be mobilized to fulfill its functions. As mentioned earlier, cut-off values are different in healthy subjects and in patients with chronic diseases. Laboratory print-outs usually show cut-off values reflecting only absolute iron deficiency in healthy individuals, somewhere in the order between 20 and 30 µg/L. Absolute iron deficiency in patients with HF may be present once serum ferritin is <100 µg/L [19], and functional iron deficiency may be present when serum ferritin is <300 µg/L with TSAT <20% [7,8,20].

## 4. Iron Deficiency in Patients with Heart Failure

Chronic HF is a cardiovascular syndrome with a high mortality, and iron deficiency has been shown to be an independent risk factor for mortality in HF [3,5]. About half of all patients with HF have either absolute iron deficiency or functional iron deficiency defined as ferritin <100 µg/L or TSAT <20% and serum ferritin 100–300 μg/L [11,21,22], respectively. This finding is only partly associated with the presence of anaemia. Indeed, many HF patients present with iron deficiency, many with anaemia, and some of these with both. Iron deficiency independently relates to exercise intolerance expressed as reduced peak oxygen uptake (peak VO_2_) and augmented ventilatory response to exercise in patients with chronic HF [8,23]. The absence of iron in the blood of patients with HF may be also reflected as reduced iron load in the bone marrow [24] and in the myocardium [25]. At the level of the bone marrow, the dominant regulatory protein that controls synthesis of new erythrocytes is erythropoietin. Erythropoietin is synthesized predominantly in the peritubular endothelium cells of the kidney [26,27] and during anaemia, caused by either reduced iron intake or blood loss, the healthy kidney can increase erythropoietin production by at least 10-fold [28].

The symptoms and signs of iron deficiency are partially explained by the presence of anaemia, but experimental evidence suggests that iron itself improves muscle function and exercise capacity in animals without changes in haemoglobin levels [29,30,31]. This finding emphasizes the role of iron as a co-factor in skeletal and cardiac muscle function.

Serum levels of hepcidin, a regulatory protein of iron metabolism synthesized by the liver, do not differ between anaemic and non-anaemic subjects [7], and there is no relationship between serum hepcidin and the change in either haemoglobin or serum C-reactive protein levels during the progression of HF. Hepcidin is an acute phase reactant whose secretion increases particularly during times of systemic inflammation. Since bacteria are dependent on the presence of iron for growth, hepcidin is meant to limit iron absorption during such episodes. Through an autocrine interaction with ferroportin, local hepcidin may protect nearby cells from iron deficiency, prevent extracellular oxidative stress, affect inflammatory responses, and deplete extracellular iron pools that are available for extracellular pathogens [32]. Hepcidin expression is regulated by body iron status, inflammation, erythroid iron demand, and hypoxia via regulation pathways involving the expression of the genes HFE, transferrin receptor 2 and hemochromatosis type 2.

The reason why iron may have an effect on HF irrespective of anaemia and haemoglobin values is that iron is an essential constituent of myoglobin, which is found in the cytoplasm and avidly binds and releases oxygen [23]. Mitochondrial function needs iron since iron is a co-factor for haem proteins that are involved in electron transfer and in ATP and energy production in the cells. In recent years, different therapeutic possibilities embrace iron replacement by oral or IV routes. The IV (IV) route is more effective than oral one, mostly as a consequence of the restricted absorption capacity in the duodenum and due to side effects of oral iron therapy that are encountered in up to 20% of all patients treated with oral iron [33,34].

## 5. Indicators of Iron Deficiency

Multiple markers are needed to better characterize iron homeostasis in HF due to the common presence of inflammation and normal total body iron stores. Serum ferritin and TSAT can be used as markers for iron deficiency and reflect body iron stores. The normal range of ferritin is 30–300 µg/L in men and 20–200 µg/L in woman and the reference range of TSAT is 16%–45%, somewhat variable between different laboratories. Cut-off values are different in patients with chronic diseases. As mentioned earlier, absolute iron deficiency is defined as serum ferritin <100 μg/L and TSAT <20%. Functional iron deficiency is defined as serum ferritin 100–300 μg/L and TSAT <20% [35]. In addition to ferritin and TSAT, measurement of soluble TfR may be a useful parameter especially to monitor erythropoiesis and in the prediction of the haematological response to iron treatment [36]. An increase in the serum value of soluble TfR reflects iron need of cells expressing it in order to improve their potential for iron uptake. An isolated rise in red cell distribution width (RDW) is one of the abnormalities often seen in anaemia and iron deficiency [37]. A low mean corpuscular volume (MCV) of erythrocytes may also be useful as diagnostic tool of absolute iron deficiency [37].

## 6. Therapies for Iron Deficiency

Different types of oral iron salts such as iron sulphate, iron fumarate, iron succinate, and iron gluconate have been used to treat iron deficiency [38,39]. The intolerance of oral iron preparations is very common as well as a number of drug interactions. Because of the numerous side effects of oral iron therapy, IV iron therapy has recently been advocated [40] including studies of the effects of different IV iron preparations on exercise tolerance in HF patients [41,42]. Available IV iron agents contain iron dextran, iron gluconate, iron sucrose, or ferric carboxymaltose. The carbohydrate portion acts as an “envelope” to prevent toxic iron reactions. Serious side effects, particularly anaphylactic reactions, are mainly driven by the carbohydrate part and have been chiefly reported for iron dextran. The newer preparations do not seem to produce anaphylactic reactions [39,41]. Interestingly, iron gluconate contains the same iron hydroxide core as iron dextran, but utilizes sucrose and gluconate to stabilize and solubilize the compound [43]. All IV iron agents are colloids that consist of iron-carbohydrate nanoparticles. The core is surrounded by a shell of carbohydrate that stabilizes the iron-oxyhydroxide, slows the release of bioactive iron, and maintains the resulting particles in colloidal suspension [44]. IV iron agents share the same core chemistry but differ from each other by the size of the core and the identity and the density of the surrounding carbohydrate [44]. A small study analyzed the effects of iron sucrose in 24 patients with chronic HF and iron deficiency compared to 11 patients with chronic HF without iron deficiency (Effect of IV Iron Sucrose on Exercise Tolerance in Anemic and Nonanemic Patients With Symptomatic Chronic Heart Failure and Iron Deficiency: FERRIC-HF). The FERRIC-HF [42] trial has shown that 16 weeks of iron sucrose therapy were well tolerated and associated with improvements in exercise capacity and symptom status in patients with HF and iron deficiency. Interestingly, these benefits were more evident in anaemic patients. Correction of the iron deficit with ferric carboxymaltose in patients with chronic HF was studied in a multicenter, double-blind, placebo-controlled trial (Ferinject assessment in patients with iron deficiency and chronic heart failure, FAIR-HF). Anker *et al.* [8] demonstrated that iron carboxymaltose treatment in iron deficient anaemic and nonanaemic patients with chronic HF increases the distance walked during the 6-min walk test, improves New York Heart Association (NYHA) class and overall quality of life. FAIR-HF enrolled 459 patients with chronic HF, both anaemic and non-anaemic (haemoglobin: 9.5–13.5 g/dL) with iron deficiency defined as ferritin <100 ng/mL or ferritin between 100 and 300 ng/mL with TSAT <20%. Interestingly, in this study, the improvement in quality of life was similar in both anaemic and non-anaemic patients, suggesting that iron deficiency represents a significant co-morbidity in chronic HF that can be treated independent of anaemia. Recently it has also been shown that patients with chronic HF and iron deficiency showed reduced health-related quality of life [45] independent of anaemia.

## 7. Clinical Implications

Current guidelines for the diagnosis and treatment of HF state that all patients should be screened for iron deficiency and anaemia, a class I recommendation based mostly on expert opinion (level of evidence: C). Patient who remain symptomatic in NYHA classes II-IV may benefit from iron supplementation, preferable via the IV route. In our clinic, we currently administer iron according to the iron deficit calculated using Ganzoni’s formula [46], *i.e*., the iron deficit in mg is calculated from the equation: body weight (kg) × [15 − actual haemoglobin (g/dL)] × 2.4 + 500. Usually, the required dose of iron is around 1000 mg, which should prompt IV administration. Only few IV iron formulations, however, can be given in high doses that permit repletion of iron stores in a reasonable time frame. Our clinical experience shows that about half of patients treated with such regimen display an increase in their peak VO_2_ value.

## 8. Conclusions and Outlook

Absolute or functional iron deficiency of total body iron is a common situation in patients with HF with and without anaemia. Iron deficiency is often accompanied by reduced exercise tolerance and reduced peak oxygen uptake. In patients with HF, untreated iron deficiency may result in anaemia and worsening NYHA class. IV iron administration may help to alleviate clinical symptoms, however, effects on mortality have not been studied so far.

Several studies with IV iron as well as with oral iron agents are currently ongoing. For example, one study aims to analyze IV iron sucrose in patients with chronic HF and chronic kidney disease (Intravenous Iron in Patients With Severe Chronic Heart Failure and Chronic Kidney Disease [47]). The aim of this trial is to assess the efficiency of IV iron therapy in the management of mild to moderate anaemia associated with chronic HF (NYHA III class) and moderate chronic kidney disease. Another ongoing study the IRON-HF [48] study aims to analyze the effects of different iron supplementation regimens (iron sucrose IV 200 mg once a week for 5 weeks or oral ferrous sulfate 200 mg twice daily for 8 weeks) on parameters of functional capacity in iron deficient patients with HF and anaemia. However, many questions remain and require further research. We look forward to future studies to answer important questions about the use of iron agents in cardiovascular diseases.
